# *Anopheles gambiae* on remote islands in the Indian Ocean: origins and prospects for malaria elimination by genetic modification of extant populations

**DOI:** 10.1038/s41598-023-44501-z

**Published:** 2023-11-27

**Authors:** Robert E. Ditter, Melina Campos, Marc W. Crepeau, João Pinto, Ali Toilibou, Yssouf Amina, Luciano Michaël Tantely, Romain Girod, Yoosook Lee, Anthony J. Cornel, Gregory C. Lanzaro

**Affiliations:** 1 Vector Genetics Laboratory, Department of Pathology, Microbiology and Immunology, UC Davis, 1089 Veterinary Medicine Dr, 4225 VM3B, Davis, CA 95616 USA; 2 Global Health and Tropical Medicine, Instituto de Higiene E Medicina Tropical, Universidade Nova de Lisboa, Lisboa, Portugal; 3 Malaria Control Program, Moroni, Comoros; 4 Medical Entomology Unit, Institut Pasteur de Madagascar, Antananarivo 101, BP1274 Ambatofotsikely, Madagascar; 5 Florida Medical Entomology Laboratory, Department of Entomology and Nematology, Institute of Food and Agricultural Sciences, University of Florida, 200 9th St SE, Vero Beach, FL 32962 USA

**Keywords:** Evolutionary genetics, Phylogenetics, Population genetics

## Abstract

The mosquito *Anopheles gambiae s.s.* is a primary malaria vector throughout sub-Saharan Africa including the islands of the Comoros archipelago (Anjouan, Grande Comore, Mayotte and Mohéli). These islands are located at the northern end of the Mozambique Channel in eastern Africa. Previous studies have shown a relatively high degree of genetic isolation between the Comoros islands and mainland populations of *A. gambiae*, but the origin of the island populations remains unclear. Here, we analyzed phylogenetic relationships among island and mainland populations using complete mitochondrial genome sequences of individual *A. gambiae* specimens. This work augments earlier studies based on analysis of the nuclear genome. We investigated the source population of *A. gambiae* for each island, estimated the number of introductions, when they occurred and explored evidence for contemporary gene flow between island and mainland populations. These studies are relevant to understanding historical patterns in the dispersal of this important malaria vector and provide information critical to assessing their potential for the exploration of genetic-based vector control methods to eliminate this disease. Phylogenetic analysis and haplotype networks were constructed from mitogenome sequences of 258 *A. gambiae* from the four islands. In addition, 112 individuals from seven countries across sub-Saharan Africa and Madagascar were included to identify potential source populations. Our results suggest that introduction events of *A. gambiae* into the Comoros archipelago were rare and recent events and support earlier claims that gene flow between the mainland and these islands is limited. This study is concordant with earlier work suggesting the suitability of these oceanic islands as appropriate sites for conducting field trial releases of genetically engineered mosquitoes (GEMs).

## Introduction

The Comoros archipelago consists of the oceanic islands of Ngazidja (Grande Comore), Mwali (Mohéli), Ndzwani (Anjouan) and Mayotte. These volcanic islands are situated in the Mozambique Channel in the Indian Ocean, approximately 300 km off the coast of Mozambique and at a similar distance from the coast of Madagascar. There are nine species of anopheline mosquitoes reported on these islands^1,2^, but *Anopheles gambiae* is the principal malaria vector^3^. Human migration to the Comoros intensified in the late nineteenth century which is correlated with the appearance of malaria transmission^4^. Since then, numerous strategies have been employed to control malaria on these islands, including mass drug administration, indoor residual spraying (IRS), and the distribution of insecticide treated bed nets^4^. However, between 2020 and 2021 the Comoros experienced a 56.95% increase in malaria cases (World Malaria Report 2022, WHO). This trend underscores the limitations of currently available methods for controlling malaria and the need for new approaches to address the malaria problem^5,6^. Among new malaria control methodologies under development are genetic based strategies that propose to modify mosquito vector populations by the introduction of exogenous parasite blocking effector genes coupled with a Cas9-based gene drive^7^.

Recent advances in genetic engineering have the promise of providing a high-impact, sustainable and cost-effective method for the elimination of malaria using genetically engineered mosquitoes (GEM) with gene drive^8^. Field trials are a key phase of the World Health Organization (WHO) Guidance Framework for evaluating and regulating GEM use^9^. This Framework states that on completion of laboratory and insectary testing (phase 1), ecologically confined field-trials (phase 2) must be conducted prior to open-field deployment (phase 3).

Oceanic islands are among the most suitable sites for field trials due to the low biodiversity and genetic complexity of resident vector populations, their genetic and geographic isolation, and their small size^10^. Previously published studies suggested a high degree of genetic isolation between *A. gambiae s.s.* populations in the Comoros and neighboring mainland populations to the west^11,12^. However, little is known about the colonization routes and patterns of introduction of this malaria vector into the islands. Here, we focused on investigating the historical movement of *A. gambiae* across Africa and into the islands using phylogenetics and mitogenome analysis. For that purpose, we analyzed complete mitochondrial genomes of *A. gambiae* collected from the Comoros Islands, Madagascar, and six continental countries. We used haplotype profiles to determine: (i) the ancestral origin of island populations, (ii) the number of introductions, (iii) an estimate of when introductions occurred, and (iv) the level of ongoing contemporary gene flow among populations.

## Results

### Mitogenome assemblies and genetic diversity

**Figure 1:**
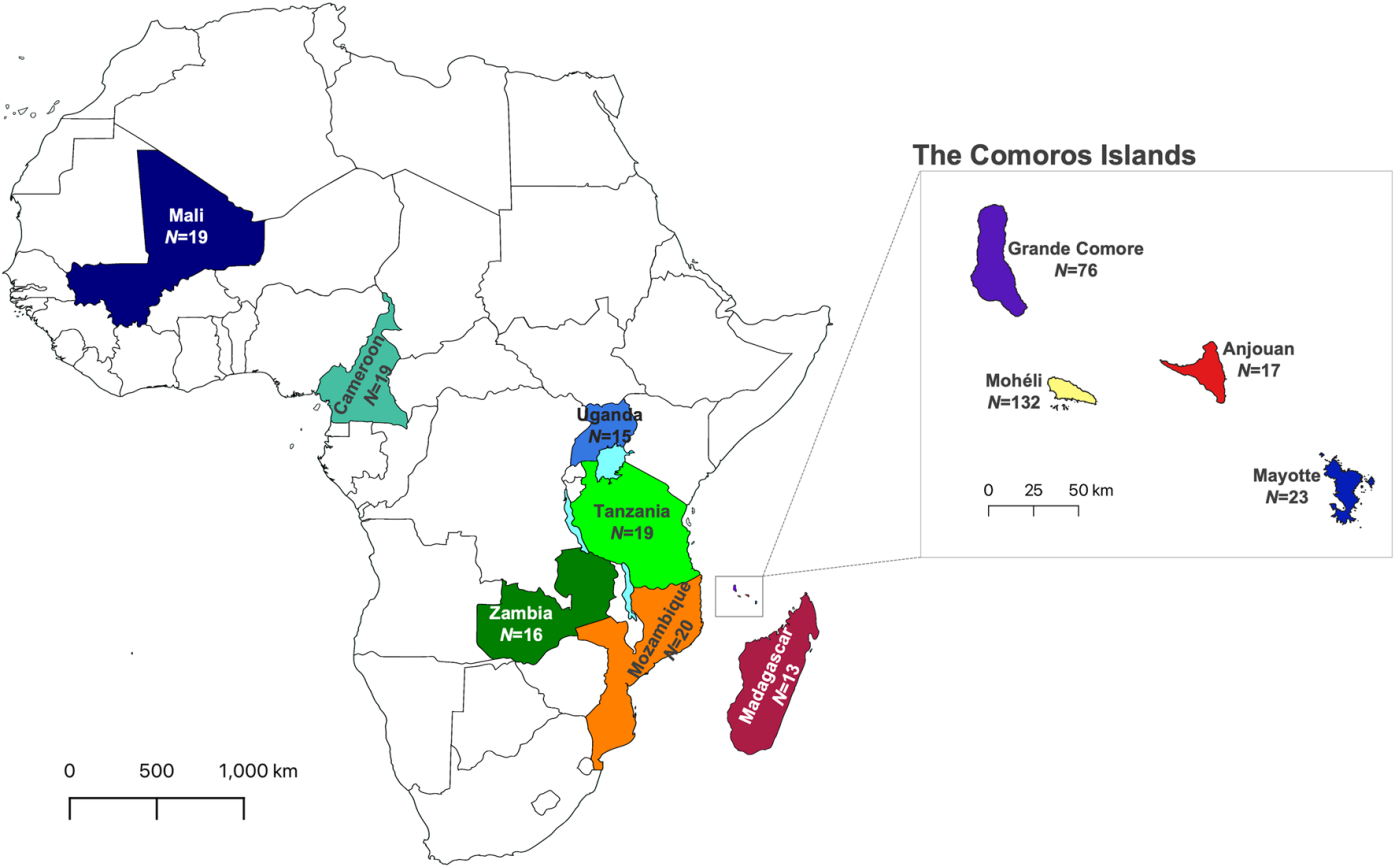
Overview of population sampling. Sampling of *Anopheles gambiae* locations in African mainland and oceanic islands. Main map includes colour-code continental countries and Madagascar. The insert map illustrates islands of the Comoros archipelago: Grande Comore, Mohéli, Anjouan and Mayotte. Sample size is displayed under each population country/island name. Maps were generated using QGIS v3.28 (available at www.qgis.org).

In total, 370 complete mitochondrial genomes were used to investigate the origin of *A. gambiae* on the islands of Anjouan, Mayotte, Mohéli and Grande Comore (Fig. 1). Potential source populations were identified from six countries across sub-Saharan Africa, including Cameroon, Mali, Mozambique, Tanzania, Uganda, and Zambia, as well as the island nation of Madagascar. New *A. gambiae* mitochondrial genome sequences were generated through whole DNA sequencing of individual mosquito specimens from Mohéli, Grande Comore, and Madagascar, as described in the methods section. Grande Comore presented the lowest nucleotide diversity (π = 1.9 × 10^–4^), despite overrepresentation in sample size, followed by Mayotte (π = 4.2 × 10^–4^), Anjouan (π = 4.3 × 10^–4^), and Mohéli (π = 4.4 × 10^–4^) (Table 1).

**Table 1:** Population genetic indexes for each population of *A. gambiae*.

Locality	*N*	V	*π*	H	H_*p*_	H_d_	k
Mali	17	186	0.00391	18	1	1.000	58.124
Cameroon	19	167	0.00303	18	1	1.000	60.000
Uganda	15	93	0.00138	15	1	1.000	20.533
Zambia	6	37	0.00107	6	1	1.000	15.867
Tanzania	18	180	0.00277	17	0.94	0.994	41.158
Mozambique	20	47	0.00050	19 (3)	0.95	0.995	7.421
Madagascar	17	57	0.00082	11 (3)	0.65	0.974	12.231
Mayotte	23	23	0.00042	15 (2)	0.65	0.917	4.791
Anjouan	17	61	0.00043	7 (4)	0.41	0.897	4.057
Mohéli	132	194	0.00044	88 (7)	0.62	0.990	6.512
Grande comore	86	59	0.00019	34 (5)	0.45	0.930	2.862

*N* sample size, *V* number of variable sites, *π* nucleotide diversity, *H* number of haplotypes (number of haplotypes after combining individuals differing by a single mutation), *H*_*p*_ sample unique haplotype proportion, *H*_*d*_ haplotype diversity, *k* mean number of mutations between haplotypes.

### Phylogenetics

**Figure 2:**
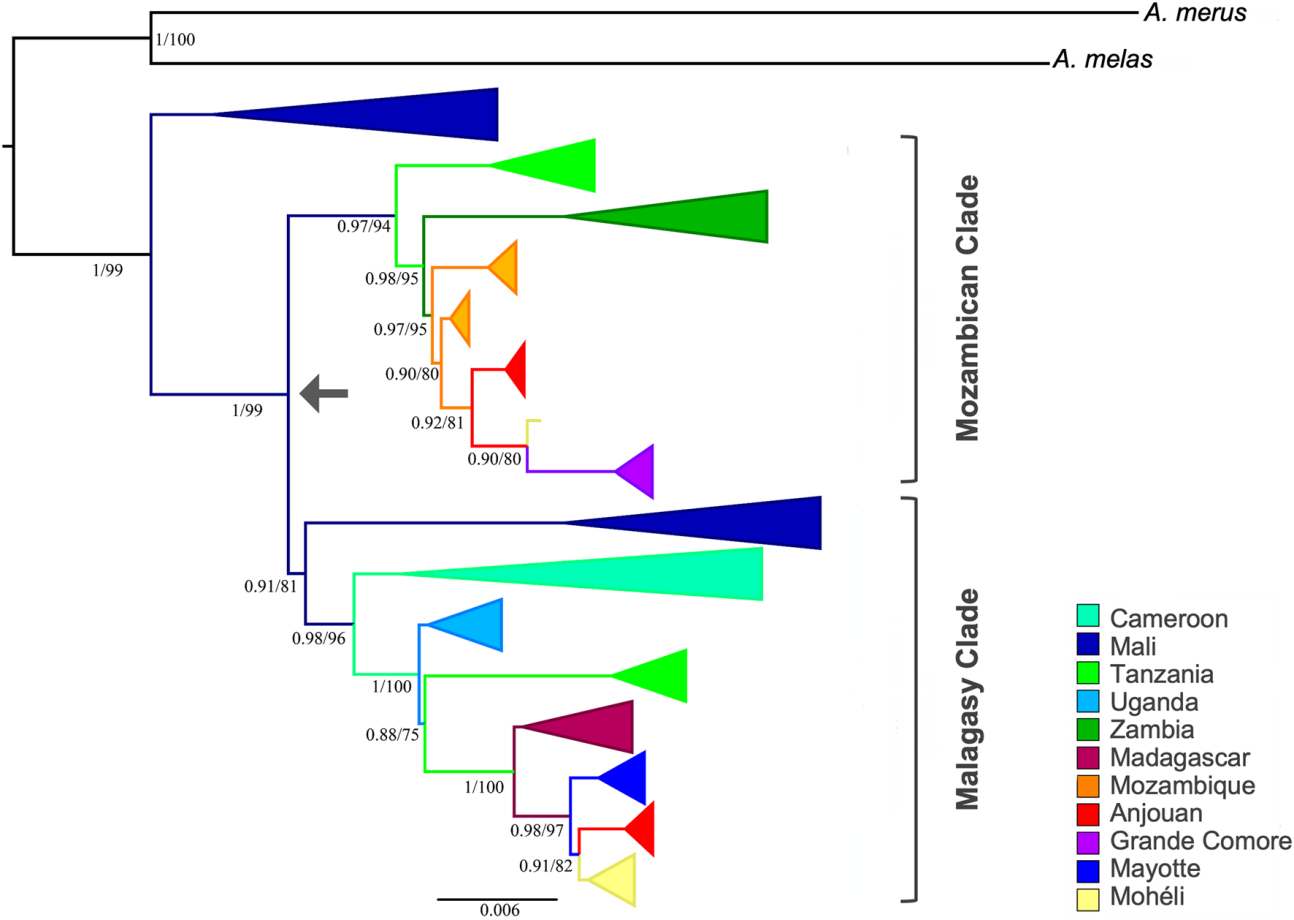
Phylogram of *Anopheles gambiae* using complete mitogenome of a subset of individuals with only unique sequences. Values > 0.7 for ML and 90% for BI are shown and represented by percentages to the left of nodes. Arrow indicates a divergence within *A. gambiae* occurring in West Africa.

The complete phylogenetic analysis represents 370 terminals of *A. gambiae s.s.* from African islands and mainland populations and additional terminals representing two sister species within the *A. gambiae* complex: *A. melas* and *A. merus* (Fig. S1). A simplified phylogenetic tree was built with one representative of each unique haplotype (Fig. 2). All relationships between localities within mitogenome phylogenies were resolved with significant support using ML and BI analysis (ML > 70 and BI > 90; Fig. 2).

Mali is recovered as the earliest branching lineage within *A. gambiae* and is sister to two clades: a Mozambican clade and a Malagasy clade. The Mozambican clade includes Tanzania, Zambia, Mozambique, Anjouan, Mohéli and Grande Comore. The Malagasy clade includes a second branch from Mali, Cameroon, Uganda, Tanzania, Madagascar, Mayotte, Anjouan, and Mohéli. Individuals from Tanzania are recovered as two distinct monophyletic clades: the Mozambican clade, as the earliest branch lineage sister to Zambia, and the Malagasy clade, preceeded by Cameroon and Uganda, and as sister to Madagascar (Fig. 2). Grande Comore is recovered as a monophyetic clade within the Mozambican clade and sister to one individual from Mohéli and monophyletic clades comprised of individuals from Anjouan and Mozambique (Fig. 2). The remaining individuals from Mohéli are recovered as a monophyletic clade within the Malagasy clade that is sister to monophyletic clades comprised of individuals from Anjouan, Mayotte and Madagascar. Both the Mozambican and Malagasy clades are recovered with strong support (Fig. 2).

**Figure 3:**
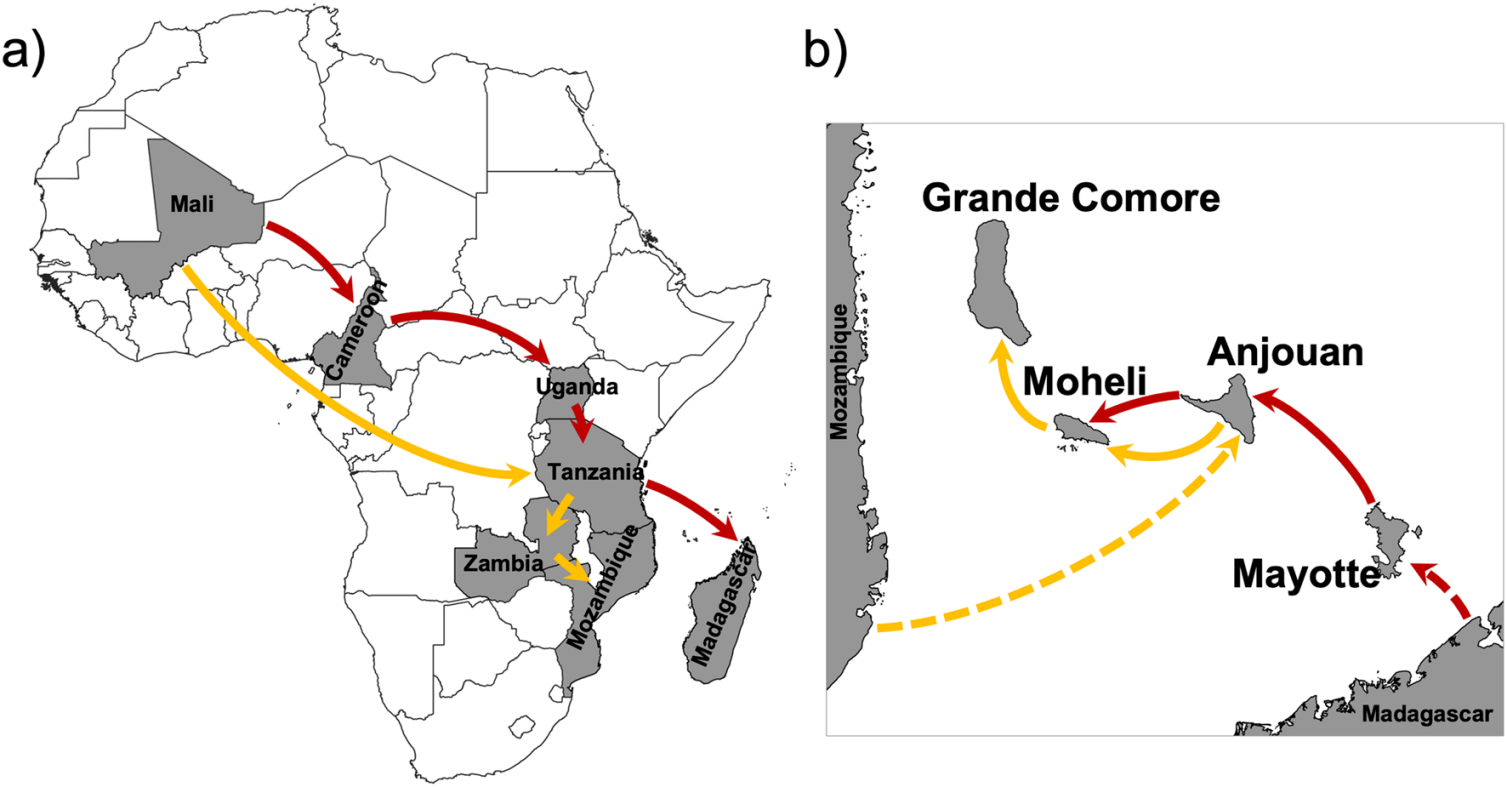
Schematic representation of the two routes of *Anopheles gambiae* dispersal from West to East Africa and subsequently into the Comoros archipelago. Arrows indicate the direction and routes of dispersal for the Malagasy clade (in red) and the Mozambican clade (in orange). (**a**) West to East dispersal of *A. gambiae* in continental Africa and Madagascar. (**b**) *A. gambiae* dispersal into the Comoros Islands. Distance between the islands and Mozambique and Madagascar not scaled. Maps were generated using QGIS v3.28 (available at www.qgis.org).

Schematic representation of the two routes of *A. gambiae* introduction from West to East Africa is shown in Fig. 3. The Malagasy clade belongs to a lineage originating in Mali and Cameroon, and first appears in Uganda in East Africa before migrating to Tanzania and then Madagascar (Fig. 3a). The Mozambican clade also belongs to a Malian lineage, but it first arrived in Tanzania in East Africa before migrating to Zambia and then Mozambique (Fig. 3a). The two haplotypes on Mayotte appear to have diverged from a single Malagasy ancestral lineage (Fig. 3b). The haplotypes in Anjouan have their origins from both the Malagasy and Mozambican clades (Fig. 3b). The majority of individuals sampled from Mohéli appear to have diverged from the Malagasy clade, but a single individual had a haplotype originating from the Mozambican clade, and they all share lineages with Anjouan. Haplotypes present in Grande Comore originate from the Mozambican clade, which appear to be diverged from a single ancestral lineage and likely originated from Mohéli (Fig. 2; Fig 3b).

### Haplotype network and migration

**Figure 4:**
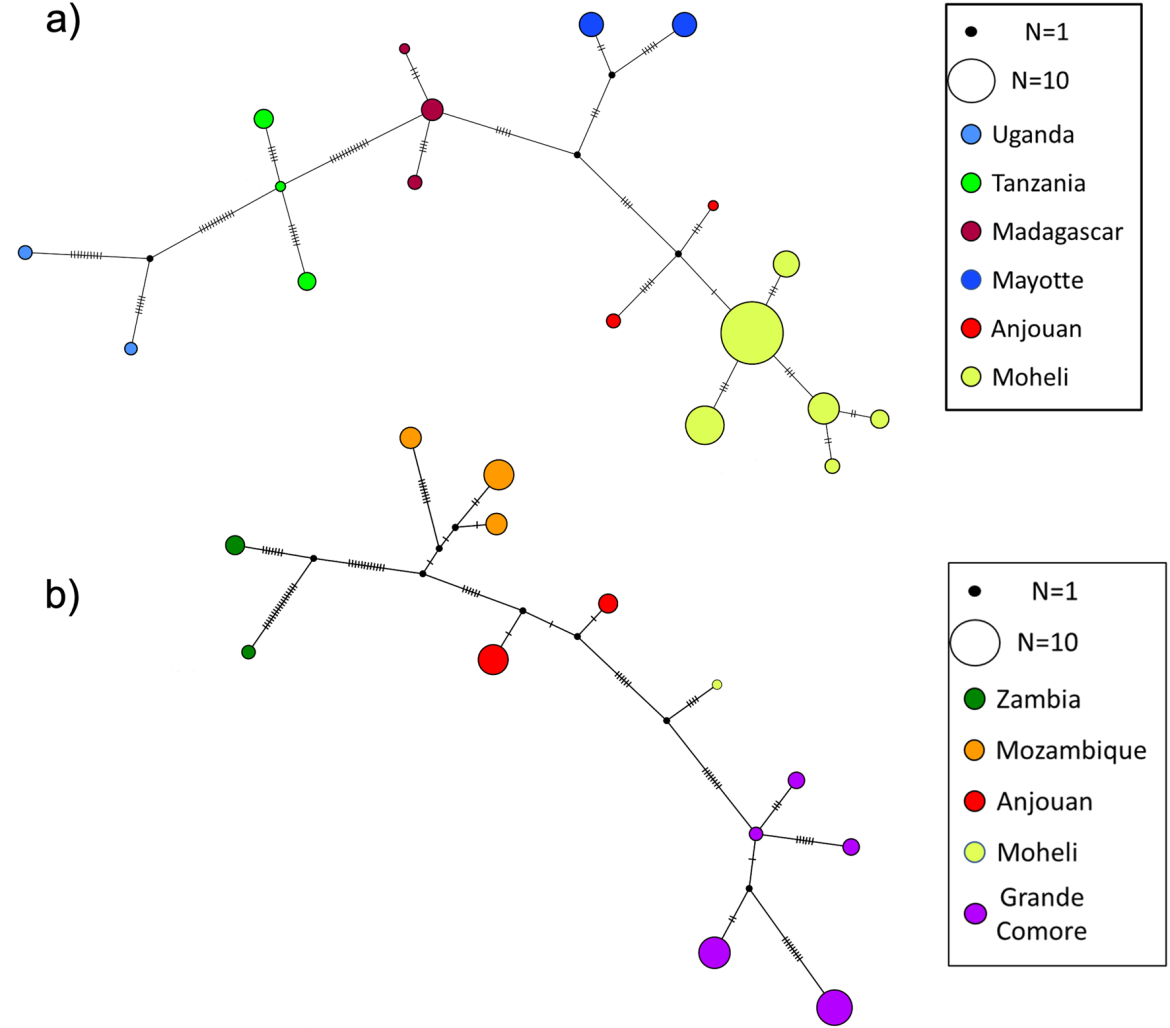
Haplotype networks of *A. gambiae* clades present in the Comoros Islands and their ancestors. (**a**) Haplotype network for the Malagasy clade. (**b**) Haplotype network for the Mozambican clade.

Haplotype network was performed separately for the two routes (Malagasy and Mozambican) of *A. gambiae* dispersal across continental Africa and into oceanic islands (Fig. 4). There are no shared haplotypes between populations of the Comoros islands and source populations represented by African mainland countries and Madagascar (Fig. 4). In the Malagasy clade route, haplotypes from Mayotte island are separated from the closest mainland haplotypes, Tanzania, by at least 20 mutations, and from Madagascar by 8 mutations (Fig. 4a). Haplotypes from Anjouan Island, in the Mozambican clade route, are seperated from Mozambique, the closest mainland haplotypes, by at least 10 mutations (Fig. 4b). In the Malagasy clade route, Anjouan island haplotypes are separated by 12 mutations from Madagascar (Fig. 4a).

**Figure 5:**
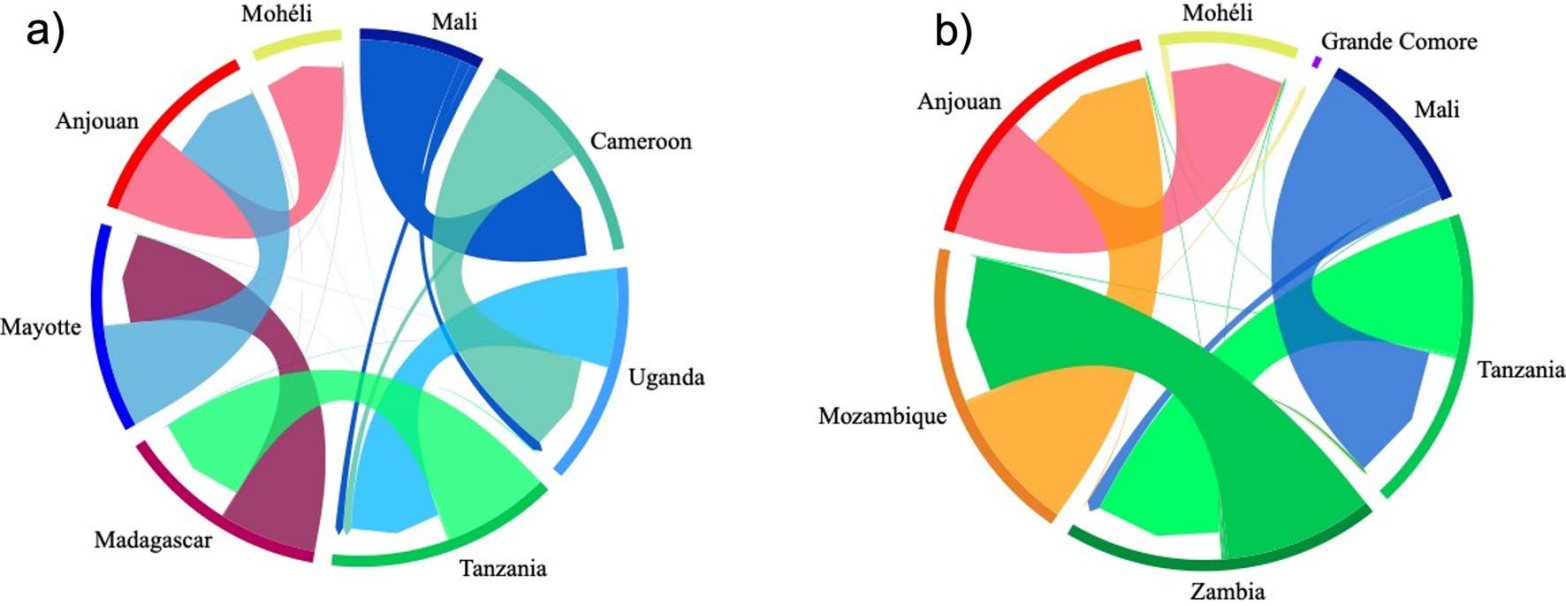
Migration pattern of *A. gambiae* populations using Migrate-n analysis. The color of each arrow corresponds to the color of the source population locality listed outside of the circle. The width of each arrow is proportional to the estimated migration rate. The arrows indicate the direction of migration, the arrow base indicates the source population and the arrowhead the recipient population. (**a**) Migration of the Malagasy clade. (**b**) Migration of the Mozambican clade.

Migrate-n analysis are illustrated as chord diagrams in Fig. 5. Migration of *A. gambiae* occurred also along two routes from West Africa to East Africa. The Malagasy route from Mali → Cameroon → Uganda → Tanzania → Madagascar → Mayotte → Anjouan → Mohéli (Fig. 5a); and the Mozambican route from Mali → Tanzania → Zambia → Mozambique → Anjouan → Mohéli → Grande Comore (Fig. 5b). Migration estimates result in similar rates of *A. gambiae* dispersal from West Africa to East Africa, and slightly decreased in rates as *A. gambiae* migrated to the islands (Fig. 5). Our analysis indicates migration along a largely unidirectional gradiatent from east to west among island populations with negligible migration occuring in the opposite direction (Fig. 5). Migration from any island population to any continental population, or to Madagascar, also appears to be highly unlikely (Fig. 5).

### Divergence time estimates

**Figure 6:**
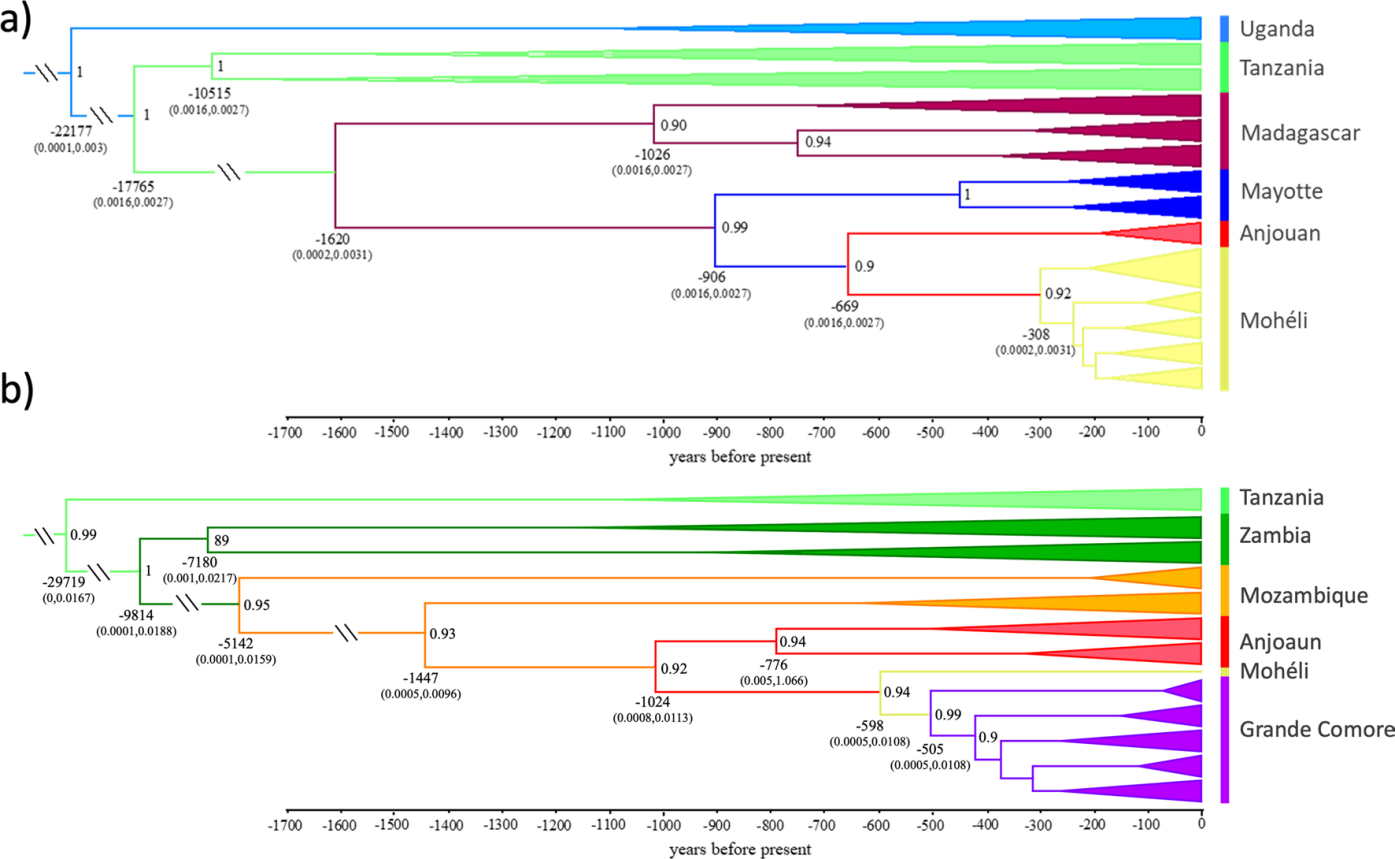
Divergence time estimates (x axis in years before present) of *Anopheles gambiae* populations from the oceanic islands, Madagascar, and mainland African populations calculated using the complete mitogenome coalescent model in BEAST2; outgroups not shown. (**a**) For the Malagasy clade. (**b**) For the Mozambican clade. Label colors correspond to haplotypes in Fig. 3. Posterior probability values > 0.9 are to the right of nodes, mean node ages are listed below nodes and the values within brackets are the 95% HPC intervals.

We recovered a well-supported phylogeny generated in BEAST2 that is concordant with ML and BI mitogenomic phylogenetic trees (Fig. 6). The annotated BEAST2 tree supports a recent divergence of populations of the four Comoros islands from ancestral mainland African populations (Fig. 6). We assumed a substitution rate of 1.87 × 10^–8^ mutations per site per year, and a generation time of three weeks. Divergence time estimates reveal that these island populations began differentiating from ancestral populations around 1600 years before present and suggest that populations on Grande Comore and Mohéli represent the most contemporary divergences begining around 600–1000 years before present respectively. Furthermore, these phylogenetic trees support introductions into Anjouan from Mayotte and Mozambique, two introductions from Anjouan into Mohéli, and a single introduction from Mohéli into Grande Comore (Fig. 6).

## Discussion

Here we report the results of an analysis of the *A. gambiae* mitogenomes among populations sampled from six continental African countries, the island of Madagascar and the four islands comprising the Comoros archipelago. The results provide evidence for two independent *A. gambiae* clades going from West to East Africa and ultimately into the Comoros Islands.

This study expands on and refines our earlier work on the origins of *A. gambiae* in the Comoros archipelago^11^. Here we have increased the number of geographic locations sampled adding sites in Tanzania and including sites in Madagascar, Mayotte, Mozambique, and Uganda, not included in our earlier study (Fig. 1). The addition of these sites revealed the second route of *A. gambiae* introduction into the Comoros islands originating from Madagascar (Fig. 4b). Our findings agree with Schmidt et al*.*^11^ on the timeline for divergence of West and East African clades, but our results support a more recent timeline of *A. gambiae* dispersal into East Africa (Fig. 6). The difference between the timeline presented here and that of Schmidt et al.^11^ is likely to be the consequence of our use of mitochondrial, rather than nuclear DNA^11,13^. When interpreting results generated by analysis of mitochondrial (mtDNA) versus nuclear DNA it is important to consider how these genomes differ, e.g., in size, mutation rate, ploidy, recombination rates and number of introns^14,15^. However, despite their smaller size, mitochondrial genomes have proven useful to the evaluation of the ancestry and demographic history of populations^16–18^.

We identified multiple haplotypes, after removing singletons, on each of the Comoros Islands as well as Madagascar and Mozambique (Table 1, Fig. 4). Haplotypes clustered into two major clades, both sharing ancestry with populations in Mali (Fig. 2). We refer to these as the Malagasy clade and the Mozambican clade with reference to the immediate ancestors of *A. gambiae* populations in the Comoros archipelago. The small number of haplotypes on each island compared to populations of *A. gambiae* in mainland Africa and the absence of haplotype divergence among subpopulations within each island is consistent with a recent colonization of the Comoros islands resulting in the observed founder effect (population bottleneck) of *A. gambiae* as previously reported^11^.

Applying a molecular clock analysis, the initial introduction into the islands of the Comoros archipelago appears to have been from Madagascar into the island of Mayotte (1.6 Ka), subsequently into Anjouan (0.9 Ka) and then Mohéli (0.7 Ka). The Malagasy clade has not arrived in Grande Comore, or if it did it has become extinct. The Mozambican clade was introduced from the west slightly later, first into Anjouan Island (1.5 Ka), subsequently moving westward into Mohéli (1.0 Ka) and finally Grande Comore (0.6 Ka). The invasion of the Comoros Islands by *A. gambiae* is almost certainly anthropogenic. Historical records suggest that *A. gambiae* was introduced into Madagascar and Anjouan during the first major wave of human migration to the islands between 500–4000ya and 1000–1100ya respectively^19–22^. Interisland dispersal (Anjouan → Mohéli, and Mohéli → Grande Comore) coincides with the establishment of trade between islands (600–1000 ya) and the arrival of the Portuguese, around 1500_AD_^4,22^. The introduction of *A. gambiae* into Grande Comore is the most recent and its introduction occurred from unidirectional migration from East to West (Fig. 5b).

Lastly, this study supports earlier work suggesting that contemporary genetic exchange between mainland Africa or Madagascar and the Comoros Islands is limited, and that these islands are well-suited for field trials of genetically engineered mosquitoes for malaria eradication^9^.

## Materials and methods

### Sampling and DNA extraction

For this study we included both newly generated and publicly available mitochondrial genome sequence data of *A. gambiae s.s.* from the Comoros Islands (Anjouan, Grande Comore, Mayotte and Mohéli), Madagascar, and six continental African countries (Fig. 1, Supplementary Table S1). The new sample set included immature stages of *A. gambiae* collected from 2 localities in Anjouan, 11 in Grande Comore, 16 in Mohéli and 4 in Madagascar. These sequences were obtained from the UC Davis Vector Genetics Laboratory (VGL) archive and from the publicly available data from the Ag1000G database (AG1000G)^23^, accession numbers are provided in Supplementary Table S1. A total of 370 samples were included in our analyses. Samples from islands included: Anjouan, *N* = 17; Grande Comore, *N* = 86; Mayotte, *N* = 23; Mohéli, *N* = 132, Madagascar *N* = 17; and samples from continental African sites were Cameroon, *N* = 19; Mali, *N* = 17; Mozambique, *N* = 20; Tanzania *N* = 18; Uganda, *N* = 15; Zambia, *N* = 6.

Whole DNA from individual mosquito specimens from the VGL archive were extracted using our established protocol on a Qiagen Biosprint^24^. Species identification was conducted using species-specific SNPs included in the DIS assay^25^. Methods used for species identification, DNA extraction and genomic sequencing of individuals from the Ag1000G database were performed as described by AG1000G (2020)^23^.

### Mitogenome sequencing and assembly

Individual library preparation was performed with 10 ng of genomic DNA as input and using KAPA HyperPlus Kit (Roche Sequencing Solutions, Indianapolis, Indiana, USA) following our protocol^26^. A dsDNA high sensitivity assay kit on a Qubit instrument (Thermo Fisher Scientific, Waltham, MA, USA) was used to quantify the initial input DNA. Library size selection (~ 500 bp) and clean-up were done with AMPure SPRI beads (Beckman Coulter Life Sciences, Indianapolis, Indiana, USA). Sequencing was performed on an Illumina HiSeq 4000 instrument at the UC Davis DNA Technologies Core facility for a pooled library of equal amounts of individual indexed libraries. Reads were demultiplexed and adapters were removed.

Raw-sequencing reads were used to assemble the mitochondria contig using NOVOPlasty version 2.6.7^27^ with 33 as K-mer’s value and default settings. Samples from the Ag1000G were downloaded as binary alignment map (BAM) files from the European Nucleotide Archive (ENA). BAM files were converted to FastQ format using BEDTools^28^ which were then used for mitochondria assembly as described above.

### Data analysis

De novo assembled mitochondrial genomes were imported into Geneious (2023.0.4) and aligned with a reference mitogenome sequence of *Anopheles gambiae* (NC_002084) acquired from GenBank. The alignment was visually inspected to confirm that no missing data, singletons, or polymorphic/ambiguities were present in the sequences. Genetic diversity indices such as nucleotide diversity (π) and haplotype diversity (*H*_*d*_) were assessed for each geographic population using DnaSP 6.12.03^29^.

### Phylogenetic analysis

The annotated mitogenome of *A. gambiae* reference was used to partition the aligned mitogenomes by coding region. A partition test of heterogeneity was conducted to determine if the complete mitogenome was appropriate to use for phylogenetic analysis, as implemented in PartitionFinder2^30^. The best fitting models of evolution for each partition were determined using PartitionFinder2. The *A. gambiae* sequence was aligned with *Anopheles* mitogenome sequences *A. melas* (KT382823), *A. merus* (NC_028220), to use as outgroups for phylogenetic analyses using MAFFT v7.450^31,32^. Maximum likelihood (ML) and Bayesian inference (BI) analyses were conducted following the method in Ditter et al.^33^. An initial phylogeny was constructed using all available sequences, followed by a simplified phylogeny that included only individuals representing unique haplotypes from all localities (*N* = 135).

### Haplotype networks and migration patterns

Unique haplotypes were identified and corrected for ambiguities and singletons using Poppr 2.0 and DnaSP 6.12.03^29,34^. A TCS haplotype network that included haplotypes was generated using POPArt 1.7^35,36^. Individuals from mainland Africa that differed by > 30 mutations from Madagascar or Mozambique were removed from dispersal route haplotype networks.

Investigation of migration between the Comoro Islands, Madagascar and mainland Africa were conducted using a Bayesian approach compiled in Migrate-n v. 5.0.3^37,38^ on the University of California Davis Genome Center High Performance Computational Cluster. Parameters were estimated under a full migration model allowing for gene flow to occur among all populations and their most recent common ancestral populations and conducted using default parameters with 4 independent runs sampling every 200 steps for 200,000 recorded steps and a burn-in of 20,000. Comparison of migration models were conducted based on their marginal likelihood and probability using thermodynamic integration with Bezier approximation as implemented in Migrate-n^39^. Migration patterns inferred from Migrate-n analyses was visualized using the Migest package in R^40^.

### Divergence time estimates

To reflect the dispersal of *A. gambiae* across Africa and into the Comoros Islands using a mitogenome-based timeline, phylogenetic trees were estimated. To this end, a multi-locus coalescent model was applied using BEAST2 v 2.6.7^41^. Intraspecific divergence times were concurrently estimated using a substitution rate of 1.87 × 10^–8^ mutations per site per year, assuming a generation time of three weeks^42,43^. Analyses were performed in triplicate on the University of California Davis Genome Center High Performance Computational cluster with Markov chains for 100 million generations or until convergence, with the first 10% of generations of each discarded as burn-in and chains sampled every 10,000 generations. As indicated by results from PartitionFinder2^30^ the HKY substation model was tested in combination with gamma site-specific rate variation and a proportion of invariant sites parameters and both a relaxed clock and a strict clock. A strict clock was selected over a relaxed clock by comparing the likelihood of results after 10,000,000 generation calibration runs. Tracer v1.7.2 was used to assess convergence of trees^44^. LogCombiner 2.6.7 was used to resample 10,000 subtrees from BEAST2 analyses, TreeAnnotator 2.6.4 was used to generate Maximum Clade Credibility trees using mean node heights, and FigTree 1.4.4 was used to visualize trees^41,45^.

### Supplementary Information

Supplementary Information.

## Data Availability

The datasets presented in this study can be found in online repositories. The names of the repository/repositories and accession number(s) can be found in Table S1. Newly sequence samples were deposited in NCBI GenBank under BioProject PRJNA971625.
