# Supplementary material for: Anopheles gambiae on remote islands in the Indian Ocean: origins and prospects for malaria elimination by genetic modification of extant populations
Source: Sci Rep. 2023 Nov 27;13:20830. doi: 10.1038/s41598-023-44501-z (PMC10682471; doi:10.1038/s41598-023-44501-z)
Supplement: Supplementary file 1 — Supplementary Information. [file 41598_2023_44501_MOESM1_ESM.pdf]

Supplementary Table 1. Metadata and accession number for individual mosquito specimen.

SampleID	BankedID	Country/Island	Lat.	Long.	Genbank no.	BioSample	BioProject
ML_BAN_383	02BANA383	Mali	12.800000	-8.050000	VGL	SAMN35108704	PRJNA971624
ML_YOR_050	02YORO0050	Mali	10.916700	-8.000000	VGL	SAMN35108707	PRJNA971624
ML_YOR_053	02YORO0053	Mali	10.916700	-8.000000	VGL	SAMN35108708	PRJNA971624
ML_YOR_054	02YORO0054	Mali	10.916700	-8.000000	VGL	SAMN35108709	PRJNA971624
ML_YOR_056	02YORO0056	Mali	10.916700	-8.000000	VGL	SAMN35108710	PRJNA971624
GC_BGI_002	0320-GC-BGI-002	Grande Comore	-11.84068	43.29272	VGL	SAMN35113286	PRJNA971625
GC_BGI_008	0320-GC-BGI-008	Grande Comore	-11.84068	43.29272	VGL	SAMN35113287	PRJNA971625
GC_BOE_076	0320-GC-BOE-076	Grande Comore	-11.56647	43.28675	VGL	SAMN35113288	PRJNA971625
GC_BOE_079	0320-GC-BOE-079	Grande Comore	-11.56618	43.28638	VGL	SAMN35113289	PRJNA971625
GC_BOE_080	0320-GC-BOE-080	Grande Comore	-11.56618	43.28638	VGL	SAMN35113290	PRJNA971625
GC_BOE_081	0320-GC-BOE-081	Grande Comore	-11.56647	43.28675	VGL	SAMN35113291	PRJNA971625
GC_BOE_084	0320-GC-BOE-084	Grande Comore	-11.56618	43.28638	VGL	SAMN35113292	PRJNA971625
GC_ORV_016	0320-GC-ORV-016	Grande Comore	-11.91337	43.49783	VGL	SAMN35113293	PRJNA971625
GC_ORV_018	0320-GC-ORV-018	Grande Comore	-11.91337	43.49783	VGL	SAMN35113294	PRJNA971625
GC_ORV_019	0320-GC-ORV-019	Grande Comore	-11.91337	43.49783	VGL	SAMN35113295	PRJNA971625
GC_ORV_025	0320-GC-ORV-025	Grande Comore	-11.91337	43.49783	VGL	SAMN35113296	PRJNA971625
GC_ORV_026	0320-GC-ORV-026	Grande Comore	-11.91337	43.49783	VGL	SAMN35113297	PRJNA971625
GC_ORV_028	0320-GC-ORV-028	Grande Comore	-11.91337	43.49783	VGL	SAMN35113298	PRJNA971625
GC_ORV_029	0320-GC-ORV-029	Grande Comore	-11.91337	43.49783	VGL	SAMN35113299	PRJNA971625
GC_ORV_041	0320-GC-ORV-041	Grande Comore	-11.91337	43.49783	VGL	SAMN35113300	PRJNA971625
GC_ORV_043	0320-GC-ORV-043	Grande Comore	-11.91363	43.49680	VGL	SAMN35113301	PRJNA971625
GC_PAN_046	0320-GC-PAN-046	Grande Comore	-11.87508	43.39520	VGL	SAMN35113302	PRJNA971625
GC_PAN_048	0320-GC-PAN-048	Grande Comore	-11.87508	43.39520	VGL	SAMN35113303	PRJNA971625
GC_PAN_050	0320-GC-PAN-050	Grande Comore	-11.87508	43.39520	VGL	SAMN35113304	PRJNA971625
GC_PAN_052	0320-GC-PAN-052	Grande Comore	-11.87508	43.39520	VGL	SAMN35113305	PRJNA971625
GC_PAN_058	0320-GC-PAN-058	Grande Comore	-11.87508	43.39520	VGL	SAMN35113306	PRJNA971625
GC_PAN_059	0320-GC-PAN-059	Grande Comore	-11.87508	43.39520	VGL	SAMN35113307	PRJNA971625
GC_PAN_060	0320-GC-PAN-060	Grande Comore	-11.87508	43.39520	VGL	SAMN35113308	PRJNA971625
GC_PAN_061	0320-GC-PAN-061	Grande Comore	-11.87508	43.39520	VGL	SAMN35113309	PRJNA971625
GC_SAL_021	0320-GC-SAL-021	Grande Comore	-11.68385	43.27503	VGL	SAMN35113310	PRJNA971625
GC_SAL_025	0320-GC-SAL-025	Grande Comore	-11.68385	43.27503	VGL	SAMN35113311	PRJNA971625
GC_SAL_029	0320-GC-SAL-029	Grande Comore	-11.68385	43.27503	VGL	SAMN35113312	PRJNA971625
GC_SAL_036	0320-GC-SAL-036	Grande Comore	-11.68385	43.27503	VGL	SAMN35113313	PRJNA971625
MO_DZZ_006	0320-MO-DZZ-006	Moheli	-12.30808	43.77463	VGL	SAMN35113314	PRJNA971625
MO_DZZ_009	0320-MO-DZZ-009	Moheli	-12.30808	43.77463	VGL	SAMN35113315	PRJNA971625
MO_DZZ_031	0320-MO-DZZ-031	Moheli	-12.30563	43.77383	VGL	SAMN35113316	PRJNA971625
MO_DZZ_032	0320-MO-DZZ-032	Moheli	-12.30563	43.77383	VGL	SAMN35113317	PRJNA971625
MO_DZZ_061	0320-MO-DZZ-061	Moheli	-12.30983	43.78257	VGL	SAMN35113318	PRJNA971625

MO_DZZ_064	0320-MO-DZZ-064	Moheli	-12.30983	43.78257	VGL	SAMN35113319	PRJNA971625
MO_FOM_001	0320-MO-FOM-001	Moheli	-12.27323	43.73438	VGL	SAMN35113320	PRJNA971625
MO_FOM_005	0320-MO-FOM-005	Moheli	-12.27323	43.73438	VGL	SAMN35113321	PRJNA971625
MO_FOM_049	0320-MO-FOM-049	Moheli	-12.27930	43.73022	VGL	SAMN35113322	PRJNA971625
MO_FOM_064	0320-MO-FOM-064	Moheli	-12.28428	43.74952	VGL	SAMN35113323	PRJNA971625
MO_FOM_069	0320-MO-FOM-069	Moheli	-12.28428	43.74952	VGL	SAMN35113324	PRJNA971625
MO_FOM_073	0320-MO-FOM-073	Moheli	-12.28428	43.74952	VGL	SAMN35113325	PRJNA971625
MO_HGN_006	0320-MO-HGN-006	Moheli	-12.34287	43.84743	VGL	SAMN35113326	PRJNA971625
MO_HGN_007	0320-MO-HGN-007	Moheli	-12.34287	43.84743	VGL	SAMN35113327	PRJNA971625
MO_HGN_009	0320-MO-HGN-009	Moheli	-12.34287	43.84743	VGL	SAMN35113328	PRJNA971625
MO_HGN_114	0320-MO-HGN-114	Moheli	-12.34000	43.84342	VGL	SAMN35113329	PRJNA971625
MO_HGN_115	0320-MO-HGN-115	Moheli	-12.34000	43.84342	VGL	SAMN35113330	PRJNA971625
MO_HGN_116	0320-MO-HGN-116	Moheli	-12.34000	43.84342	VGL	SAMN35113331	PRJNA971625
MO_HMV_004	0320-MO-HMV-004	Moheli	-12.38158	43.83900	VGL	SAMN35113332	PRJNA971625
MO_HMV_006	0320-MO-HMV-006	Moheli	-12.38158	43.83900	VGL	SAMN35113333	PRJNA971625
MO_HMV_043	0320-MO-HMV-043	Moheli	-12.38087	43.83940	VGL	SAMN35113334	PRJNA971625
MO_HMV_091	0320-MO-HMV-091	Moheli	-12.37698	43.83893	VGL	SAMN35113335	PRJNA971625
MO_HMV_092	0320-MO-HMV-092	Moheli	-12.37698	43.83893	VGL	SAMN35113336	PRJNA971625
MO_HMV_093	0320-MO-HMV-093	Moheli	-12.37698	43.83893	VGL	SAMN35113337	PRJNA971625
MO_ITM_004	0320-MO-ITM-004	Moheli	-12.36913	43.86848	VGL	SAMN35113338	PRJNA971625
MO_ITM_005	0320-MO-ITM-005	Moheli	-12.36913	43.86848	VGL	SAMN35113339	PRJNA971625
MO_ITM_033	0320-MO-ITM-033	Moheli	-12.36913	43.86848	VGL	SAMN35113340	PRJNA971625
MO_ITM_034	0320-MO-ITM-034	Moheli	-12.36913	43.86848	VGL	SAMN35113341	PRJNA971625
MO_ITM_061	0320-MO-ITM-061	Moheli	-12.37048	43.86775	VGL	SAMN35113342	PRJNA971625
MO_ITM_062	0320-MO-ITM-062	Moheli	-12.37048	43.86775	VGL	SAMN35113343	PRJNA971625
MO_KGN_003	0320-MO-KGN-003	Moheli	-12.351567	43.822283	VGL	SAMN35113344	PRJNA971625
MO_KGN_006	0320-MO-KGN-006	Moheli	-12.35157	43.82228	VGL	SAMN35113345	PRJNA971625
MO_KGN_007	0320-MO-KGN-007	Moheli	-12.35157	43.82228	VGL	SAMN35113346	PRJNA971625
MO_KGN_017	0320-MO-KGN-017	Moheli	-12.35157	43.82228	VGL	SAMN35113347	PRJNA971625
MO_KGN_019	0320-MO-KGN-019	Moheli	-12.35157	43.82228	VGL	SAMN35113348	PRJNA971625
MO_KGN_020	0320-MO-KGN-020	Moheli	-12.35157	43.82228	VGL	SAMN35113349	PRJNA971625
MO_MBT_014	0320-MO-MBT-014	Moheli	-12.26433	43.69717	VGL	SAMN35113350	PRJNA971625
MO_MBT_017	0320-MO-MBT-017	Moheli	-12.26433	43.69717	VGL	SAMN35113351	PRJNA971625
MO_MBT_018	0320-MO-MBT-018	Moheli	-12.26433	43.69717	VGL	SAMN35113352	PRJNA971625
MO_MBT_019	0320-MO-MBT-019	Moheli	-12.26433	43.69717	VGL	SAMN35113353	PRJNA971625
MO_MBT_032	0320-MO-MBT-032	Moheli	-12.26433	43.69717	VGL	SAMN35113354	PRJNA971625
MO_MBT_034	0320-MO-MBT-034	Moheli	-12.26433	43.69717	VGL	SAMN35113355	PRJNA971625
MO_MBT_061	0320-MO-MBT-061	Moheli	-12.28210	43.69983	VGL	SAMN35113356	PRJNA971625
MO_MBT_065	0320-MO-MBT-065	Moheli	-12.28210	43.69983	VGL	SAMN35113357	PRJNA971625
MO_MBT_066	0320-MO-MBT-066	Moheli	-12.28210	43.69983	VGL	SAMN35113358	PRJNA971625
MO_MBT_067	0320-MO-MBT-067	Moheli	-12.28210	43.69983	VGL	SAMN35113359	PRJNA971625
MO_MBT_069	0320-MO-MBT-069	Moheli	-12.28210	43.69983	VGL	SAMN35113360	PRJNA971625

MO_MBT_071	0320-MO-MBT-071	Moheli	-12.28210	43.69983	VGL	SAMN35113361	PRJNA971625
MO_NDD_051	0320-MO-NDD-051	Moheli	-12.35220	43.67963	VGL	SAMN35113362	PRJNA971625
MO_NDD_063	0320-MO-NDD-063	Moheli	-12.33568	43.68398	VGL	SAMN35113363	PRJNA971625
MO_NDD_072	0320-MO-NDD-072	Moheli	-12.33568	43.68398	VGL	SAMN35113364	PRJNA971625
MO_NDD_081	0320-MO-NDD-081	Moheli	-12.33568	43.68398	VGL	SAMN35113365	PRJNA971625
MO_NDD_082	0320-MO-NDD-082	Moheli	-12.33568	43.68398	VGL	SAMN35113366	PRJNA971625
MO_NDD_083	0320-MO-NDD-083	Moheli	-12.33568	43.68398	VGL	SAMN35113367	PRJNA971625
MO_NDR_013	0320-MO-NDR-013	Moheli	-12.35707	43.75355	VGL	SAMN35113368	PRJNA971625
MO_NDR_036	0320-MO-NDR-036	Moheli	-12.35358	43.75183	VGL	SAMN35113369	PRJNA971625
MO_NDR_039	0320-MO-NDR-039	Moheli	-12.35358	43.75183	VGL	SAMN35113370	PRJNA971625
MO_NDR_042	0320-MO-NDR-042	Moheli	-12.35358	43.75183	VGL	SAMN35113371	PRJNA971625
MO_NDR_045	0320-MO-NDR-045	Moheli	-12.35358	43.75183	VGL	SAMN35113372	PRJNA971625
MO_NDR_050	0320-MO-NDR-050	Moheli	-12.35358	43.75183	VGL	SAMN35113373	PRJNA971625
MO_SZI_004	0320-MO-SZI-004	Moheli	-12.33980	43.78183	VGL	SAMN35113374	PRJNA971625
MO_SZI_005	0320-MO-SZI-005	Moheli	-12.33980	43.78183	VGL	SAMN35113375	PRJNA971625
MO_SZI_006	0320-MO-SZI-006	Moheli	-12.33980	43.78183	VGL	SAMN35113376	PRJNA971625
MO_SZI_008	0320-MO-SZI-008	Moheli	-12.33980	43.78183	VGL	SAMN35113377	PRJNA971625
MO_SZI_009	0320-MO-SZI-009	Moheli	-12.33980	43.78183	VGL	SAMN35113378	PRJNA971625
MO_SZI_010	0320-MO-SZI-010	Moheli	-12.33980	43.78183	VGL	SAMN35113379	PRJNA971625
MO_WAN_005	0320-MO-WAN-005	Moheli	-12.34380	43.80030	VGL	SAMN35113380	PRJNA971625
MO_WAN_010	0320-MO-WAN-010	Moheli	-12.34380	43.80030	VGL	SAMN35113381	PRJNA971625
MO_WAN_018	0320-MO-WAN-018	Moheli	-12.34380	43.80030	VGL	SAMN35113382	PRJNA971625
MO_WAN_031	0320-MO-WAN-031	Moheli	-12.34380	43.80027	VGL	SAMN35113383	PRJNA971625
MO_WAN_032	0320-MO-WAN-032	Moheli	-12.34380	43.80027	VGL	SAMN35113384	PRJNA971625
MO_WAN_034	0320-MO-WAN-034	Moheli	-12.34380	43.80027	VGL	SAMN35113385	PRJNA971625
ML_DON_022	06DONE0022	Mali	12.80683	-7.98476	VGL	SAMN35108705	PRJNA590708
ML_DON_045	06DONE0045	Mali	12.80683	-7.984760	VGL	SAMN35108707	PRJNA590708
ML_DON_046	06DONE0046	Mali	12.80683	-7.984760	VGL	SAMN35108708	PRJNA590708
ML_DON_022	06DONE022	Mali	12.80683	-7.984760	VGL	SAMN35108705	PRJNA971624
ML_SEL_102	06SELI0102	Mali	11.700000	-8.283300	VGL	SAMN35108706	PRJNA971624
ML_SEL_114	06SELI0114	Mali	11.700000	-8.283300	VGL	SAMN35108710	PRJNA590708
ML_SEL_115	06SELI0115	Mali	11.700000	-8.283300	VGL	SAMN19188256	PRJNA590708
ML_BAN_021	1992BANA021	Mali	12.800000	-8.050000	VGL	SAMN35108699	PRJNA971624
ML_BAN_024	1992BANA024	Mali	12.800000	-8.050000	VGL	SAMN35108700	PRJNA971624
AN_ASS_042	2011ASS042	Anjouan	-12.23727	44.31655	VGL	SAMN35113386	PRJNA971625
AN_ASS_097	2011ASS097	Anjouan	-12.23727	44.31655	VGL	SAMN13337418	PRJNA590708
AN_ASS_173	2011ASS173	Anjouan	-12.23727	44.31655	VGL	SAMN35113387	PRJNA971625
AN_ASS_175	2011ASS175	Anjouan	-12.23727	44.31655	VGL	SAMN35113388	PRJNA971625
AN_ASS_195	2011ASS195	Anjouan	-12.23727	44.31655	VGL	SAMN13337419	PRJNA590708
AN_ASS_211	2011ASS211	Anjouan	-12.23727	44.31655	VGL	SAMN35113389	PRJNA971625
AN_ASS_213	2011ASS213	Anjouan	-12.23727	44.31655	VGL	SAMN13337420	PRJNA590708
GC_BOE_005	2011BOE005	Grande Comore	-11.56618	43.28638	VGL	SAMN13337378	PRJNA590708

GC_BOE_006	2011BOE006	Grande Comore	-11.56618	43.28638	VGL	SAMN35113390	PRJNA971625
GC_BOE_007	2011BOE007	Grande Comore	-11.56618	43.28638	VGL	SAMN13337379	PRJNA590708
GC_BOE_008	2011BOE008	Grande Comore	-11.56618	43.28638	VGL	SAMN35113391	PRJNA971625
GC_BOE_015	2011BOE015	Grande Comore	-11.56618	43.28638	VGL	SAMN35113392	PRJNA971625
GC_BOE_018	2011BOE018	Grande Comore	-11.56618	43.28638	VGL	SAMN35113393	PRJNA971625
GC_BOE_020	2011BOE020	Grande Comore	-11.56618	43.28638	VGL	SAMN13337380	PRJNA590708
GC_BOE_035	2011BOE035	Grande Comore	-11.56618	43.28638	VGL	SAMN13337381	PRJNA590708
GC_BOE_036	2011BOE036	Grande Comore	-11.56618	43.28638	VGL	SAMN13337382	PRJNA590708
GC_BOE_048	2011BOE048	Grande Comore	-11.56618	43.28638	VGL	SAMN13337383	PRJNA590708
GC_BOE_068	2011BOE068	Grande Comore	-11.56618	43.28638	VGL	SAMN35113394	PRJNA971625
GC_BOU_003	2011BOU003	Grande Comore	-11.48943	43.39748	VGL	SAMN13337372	PRJNA590708
GC_BOU_012	2011BOU012	Grande Comore	-11.48943	43.39748	VGL	SAMN35113395	PRJNA971625
GC_BOU_015	2011BOU015	Grande Comore	-11.48943	43.39748	VGL	SAMN13337373	PRJNA590708
GC_BOU_016	2011BOU016	Grande Comore	-11.48943	43.39748	VGL	SAMN35113396	PRJNA971625
GC_BOU_018	2011BOU018	Grande Comore	-11.48943	43.39748	VGL	SAMN35113397	PRJNA971625
GC_BOU_019	2011BOU019	Grande Comore	-11.48943	43.39748	VGL	SAMN13337374	PRJNA590708
GC_BOU_028	2011BOU028	Grande Comore	-11.48943	43.39748	VGL	SAMN35113398	PRJNA971625
GC_BOU_029	2011BOU029	Grande Comore	-11.48943	43.39748	VGL	SAMN13337375	PRJNA590708
GC_BOU_030	2011BOU030	Grande Comore	-11.48943	43.39748	VGL	SAMN13337376	PRJNA590708
GC_BOU_042	2011BOU042	Grande Comore	-11.48943	43.39748	VGL	SAMN13337377	PRJNA590708
MO_FOM_002	2011FOM002	Moheli	-12.27690	43.73148	VGL	SAMN35113399	PRJNA971625
MO_FOM_004	2011FOM004	Moheli	-12.27690	43.73148	VGL	SAMN35113400	PRJNA971625
MO_FOM_005_11	2011FOM005	Moheli	-12.27690	43.73148	VGL	SAMN35113401	PRJNA971625
MO_FOM_012	2011FOM012	Moheli	-12.27690	43.73148	VGL	SAMN35113402	PRJNA971625
MO_FOM_013	2011FOM013	Moheli	-12.27690	43.73148	VGL	SAMN35113403	PRJNA971625
MO_FOM_017	2011FOM017	Moheli	-12.27690	43.73148	VGL	SAMN35113404	PRJNA971625
MO_FOM_019	2011FOM019	Moheli	-12.27690	43.73148	VGL	SAMN35113405	PRJNA971625
MO_FOM_021	2011FOM021	Moheli	-12.27690	43.73148	VGL	SAMN35113406	PRJNA971625
MO_FOM_028	2011FOM028	Moheli	-12.27690	43.73148	VGL	SAMN35113407	PRJNA971625
MO_FOM_034	2011FOM034	Moheli	-12.27690	43.73148	VGL	SAMN35113408	PRJNA971625
MO_HOA_001	2011HOA001	Moheli	-12.25742	43.67292	VGL	SAMN35113409	PRJNA971625
MO_HOA_002	2011HOA002	Moheli	-12.25742	43.67292	VGL	SAMN35113410	PRJNA971625
MO_HOA_004	2011HOA004	Moheli	-12.25742	43.67292	VGL	SAMN35113411	PRJNA971625
MO_HOA_009	2011HOA009	Moheli	-12.25742	43.67292	VGL	SAMN35113412	PRJNA971625
MO_HOA_011	2011HOA011	Moheli	-12.25742	43.67292	VGL	SAMN35113413	PRJNA971625
MO_HOA_013	2011HOA013	Moheli	-12.25742	43.67292	VGL	SAMN35113414	PRJNA971625
MO_HOA_024	2011HOA024	Moheli	-12.25742	43.67292	VGL	SAMN35113415	PRJNA971625
MO_HOA_025	2011HOA025	Moheli	-12.25742	43.67292	VGL	SAMN35113416	PRJNA971625
MO_HOA_032	2011HOA032	Moheli	-12.25742	43.67292	VGL	SAMN35113417	PRJNA971625
MO_HOA_053	2011HOA053	Moheli	-12.25742	43.67292	VGL	SAMN35113418	PRJNA971625
GC_MAL_002	2011MAL002	Grande Comore	-11.88647	43.50628	VGL	SAMN35113419	PRJNA971625
GC_MAL_005	2011MAL005	Grande Comore	-11.88647	43.50628	VGL	SAMN35113420	PRJNA971625

GC_MAL_006	2011MAL006	Grande Comore	-11.88647	43.50628	VGL	SAMN13337400	PRJNA590708
GC_MAL_009	2011MAL009	Grande Comore	-11.88647	43.50628	VGL	SAMN35113421	PRJNA971625
GC_MAL_011	2011MAL011	Grande Comore	-11.88647	43.50628	VGL	SAMN35113422	PRJNA971625
GC_MAL_017	2011MAL017	Grande Comore	-11.88647	43.50628	VGL	SAMN13337401	PRJNA590708
GC_MAL_020	2011MAL020	Grande Comore	-11.88647	43.50628	VGL	SAMN13337402	PRJNA590708
GC_MAL_028	2011MAL028	Grande Comore	-11.88647	43.50628	VGL	SAMN13337403	PRJNA590708
GC_MAL_083	2011MAL083	Grande Comore	-11.88647	43.50628	VGL	SAMN13337404	PRJNA590708
GC_MAL_088	2011MAL088	Grande Comore	-11.88647	43.50628	VGL	SAMN13337405	PRJNA590708
MO_MIR_006	2011MIR006	Moheli	-12.30200	43.63720	VGL	SAMN35113423	PRJNA971625
MO_MIR_007	2011MIR007	Moheli	-12.30200	43.63720	VGL	SAMN35113424	PRJNA971625
MO_MIR_009	2011MIR009	Moheli	-12.30200	43.63720	VGL	SAMN35113425	PRJNA971625
MO_MIR_014	2011MIR014	Moheli	-12.30200	43.63720	VGL	SAMN35113426	PRJNA971625
MO_MIR_019	2011MIR019	Moheli	-12.30200	43.63720	VGL	SAMN13337406	PRJNA590708
MO_MIR_032	2011MIR032	Moheli	-12.30200	43.63720	VGL	SAMN13337407	PRJNA590708
MO_MIR_033	2011MIR033	Moheli	-12.30200	43.63720	VGL	SAMN13337408	PRJNA590708
MO_MIR_036	2011MIR036	Moheli	-12.30200	43.63720	VGL	SAMN13337409	PRJNA590708
MO_MIR_056	2011MIR056	Moheli	-12.30200	43.63720	VGL	SAMN13337410	PRJNA590708
MO_MIR_058	2011MIR058	Moheli	-12.30200	43.63720	VGL	SAMN13337411	PRJNA590708
AN_MOY_057	2011MOY057	Anjouan	-12.30927	44.43951	VGL	SAMN13337421	PRJNA590708
AN_MOY_061	2011MOY061	Anjouan	-12.30927	44.43951	VGL	SAMN35113427	PRJNA971625
AN_MOY_063	2011MOY063	Anjouan	-12.30927	44.43951	VGL	SAMN13337422	PRJNA590708
AN_MOY_069	2011MOY069	Anjouan	-12.30927	44.43951	VGL	SAMN13337423	PRJNA590708
AN_MOY_072	2011MOY072	Anjouan	-12.30927	44.43951	VGL	SAMN13337424	PRJNA590708
AN_MOY_073	2011MOY073	Anjouan	-12.30927	44.43951	VGL	SAMN13337425	PRJNA590708
AN_MOY_149	2011MOY149	Anjouan	-12.30927	44.43951	VGL	SAMN35113428	PRJNA971625
AN_MOY_155	2011MOY155	Anjouan	-12.30927	44.43951	VGL	SAMN35113429	PRJNA971625
AN_MOY_158	2011MOY158	Anjouan	-12.30927	44.43951	VGL	SAMN35113430	PRJNA971625
AN_MOY_159	2011MOY159	Anjouan	-12.30927	44.43951	VGL	SAMN35113431	PRJNA971625
GC_MUT_002	2011MUT002	Grande Comore	-11.60992	43.39032	VGL	SAMN13337390	PRJNA590708
GC_MUT_012	2011MUT012	Grande Comore	-11.60992	43.39032	VGL	SAMN35113432	PRJNA971625
GC_MUT_020	2011MUT020	Grande Comore	-11.60992	43.39032	VGL	SAMN35113433	PRJNA971625
GC_MUT_022	2011MUT022	Grande Comore	-11.60992	43.39032	VGL	SAMN35113434	PRJNA971625
GC_MUT_031	2011MUT031	Grande Comore	-11.60992	43.39032	VGL	SAMN35113435	PRJNA971625
GC_MUT_038	2011MUT038	Grande Comore	-11.60992	43.39032	VGL	SAMN13337391	PRJNA590708
GC_MUT_039	2011MUT039	Grande Comore	-11.60992	43.39032	VGL	SAMN13337392	PRJNA590708
GC_MUT_041	2011MUT041	Grande Comore	-11.60992	43.39032	VGL	SAMN13337393	PRJNA590708
GC_MUT_045	2011MUT045	Grande Comore	-11.60992	43.39032	VGL	SAMN13337394	PRJNA590708
GC_MUT_046	2011MUT046	Grande Comore	-11.60992	43.39032	VGL	SAMN13337395	PRJNA590708
MO_NDR_001	2011NDR001	Moheli	-12.35487	43.75080	VGL	SAMN35113436	PRJNA971625
MO_NDR_003	2011NDR003	Moheli	-12.35487	43.75080	VGL	SAMN35113437	PRJNA971625
MO_NDR_004	2011NDR004	Moheli	-12.35487	43.75080	VGL	SAMN35113438	PRJNA971625
MO_NDR_006	2011NDR006	Moheli	-12.35487	43.75080	VGL	SAMN35113439	PRJNA971625

MO_NDR_009	2011NDR009	Moheli	-12.35487	43.75080	VGL	SAMN35113440	PRJNA971625
MO_NDR_016	2011NDR016	Moheli	-12.35487	43.75080	VGL	SAMN35113441	PRJNA971625
MO_NDR_017	2011NDR017	Moheli	-12.35487	43.75080	VGL	SAMN35113442	PRJNA971625
MO_NDR_020	2011NDR020	Moheli	-12.35487	43.75080	VGL	SAMN35113443	PRJNA971625
MO_NDR_033	2011NDR033	Moheli	-12.35487	43.75080	VGL	SAMN35113444	PRJNA971625
MO_NDR_042_011	2011NDR042	Moheli	-12.35487	43.75080	VGL	SAMN35113445	PRJNA971625
GC_OSS_001	2011OSS001	Grande Comore	-11.58842	43.27763	VGL	SAMN13337384	PRJNA590708
GC_OSS_005	2011OSS005	Grande Comore	-11.58842	43.27763	VGL	SAMN35113446	PRJNA971625
GC_OSS_008	2011OSS008	Grande Comore	-11.58842	43.27763	VGL	SAMN35113447	PRJNA971625
GC_OSS_012	2011OSS012	Grande Comore	-11.58842	43.27763	VGL	SAMN13337385	PRJNA590708
GC_OSS_013	2011OSS013	Grande Comore	-11.58842	43.27763	VGL	SAMN13337386	PRJNA590708
GC_OSS_017	2011OSS017	Grande Comore	-11.58842	43.27763	VGL	SAMN13337387	PRJNA590708
GC_OSS_018	2011OSS018	Grande Comore	-11.58842	43.27763	VGL	SAMN13337388	PRJNA590708
GC_OSS_020	2011OSS020	Grande Comore	-11.58842	43.27763	VGL	SAMN35113448	PRJNA971625
GC_OSS_027	2011OSS027	Grande Comore	-11.58842	43.27763	VGL	SAMN13337389	PRJNA590708
GC_SAL_001	2011SAL001	Grande Comore	-11.68030	43.26610	VGL	SAMN13337396	PRJNA590708
GC_SAL_002	2011SAL002	Grande Comore	-11.68030	43.26610	VGL	SAMN35113449	PRJNA971625
GC_SAL_003	2011SAL003	Grande Comore	-11.68030	43.26610	VGL	SAMN13337397	PRJNA590708
GC_SAL_004	2011SAL004	Grande Comore	-11.68030	43.26610	VGL	SAMN35113450	PRJNA971625
GC_SAL_005	2011SAL005	Grande Comore	-11.68030	43.26610	VGL	SAMN13337398	PRJNA590708
GC_SAL_006	2011SAL006	Grande Comore	-11.68030	43.26610	VGL	SAMN35113451	PRJNA971625
GC_SAL_007	2011SAL007	Grande Comore	-11.68030	43.26610	VGL	SAMN35113452	PRJNA971625
GC_SAL_008	2011SAL008	Grande Comore	-11.68030	43.26610	VGL	SAMN13337399	PRJNA590708
MO_WAL_001	2011WAL001	Moheli	-12.33825	43.66882	VGL	SAMN35113453	PRJNA971625
MO_WAL_002	2011WAL002	Moheli	-12.33825	43.66882	VGL	SAMN35113454	PRJNA971625
MO_WAL_006	2011WAL006	Moheli	-12.33825	43.66882	VGL	SAMN35113455	PRJNA971625
MO_WAL_008	2011WAL008	Moheli	-12.33825	43.66882	VGL	SAMN35113456	PRJNA971625
MO_WAL_027	2011WAL027	Moheli	-12.33825	43.66882	VGL	SAMN35113457	PRJNA971625
MO_WAL_036	2011WAL036	Moheli	-12.33825	43.66882	VGL	SAMN35113458	PRJNA971625
MO_WAL_039	2011WAL039	Moheli	-12.33825	43.66882	VGL	SAMN35113459	PRJNA971625
MO_WAL_041	2011WAL041	Moheli	-12.33825	43.66882	VGL	SAMN35113460	PRJNA971625
MO_WAL_050	2011WAL050	Moheli	-12.33825	43.66882	VGL	SAMN35113461	PRJNA971625
MO_WAL_054	2011WAL054	Moheli	-12.33825	43.66882	VGL	SAMN35113462	PRJNA971625
MO_WAN_003	2011WAN003	Moheli	-12.34510	43.80010	VGL	SAMN35113463	PRJNA971625
MO_WAN_004	2011WAN004	Moheli	-12.34510	43.80010	VGL	SAMN35113464	PRJNA971625
MO_WAN_006	2011WAN006	Moheli	-12.34510	43.80010	VGL	SAMN35113465	PRJNA971625
MO_WAN_011	2011WAN011	Moheli	-12.34510	43.80010	VGL	SAMN35113466	PRJNA971625
MO_WAN_033	2011WAN033	Moheli	-12.34510	43.80010	VGL	SAMN13337412	PRJNA590708
MO_WAN_035	2011WAN035	Moheli	-12.34510	43.80010	VGL	SAMN13337413	PRJNA590708
MO_WAN_041	2011WAN041	Moheli	-12.34510	43.80010	VGL	SAMN13337414	PRJNA590708
MO_WAN_042	2011WAN042	Moheli	-12.34510	43.80010	VGL	SAMN13337415	PRJNA590708
MO_WAN_043	2011WAN043	Moheli	-12.34510	43.80010	VGL	SAMN13337416	PRJNA590708

MO_WAN_045	2011WAN045	Moheli	-12.34510	43.80010	VGL	SAMN13337417	PRJNA590708
TZ_ZK_039	2012TNZK039	Tanzania	-6.83330	39.26670	VGL	SAMN13337361	PRJNA590708
TZ_ZK_042	2012TNZK042	Tanzania	-6.83330	39.26670	VGL	SAMN13337362	PRJNA590708
TZ_ZK_044	2012TNZK044	Tanzania	-6.83330	39.26670	VGL	SAMN13337363	PRJNA590708
TZ_ZK_048	2012TNZK048	Tanzania	-6.83330	39.26670	VGL	SAMN13337364	PRJNA590708
TZ_ZK_057	2012TNZK057	Tanzania	-6.83330	39.26670	VGL	SAMN13337365	PRJNA590708
ZA_NCHE_01	2015NCHE001	Zambia	-9.33300	28.71670	VGL	SAMN13337366	PRJNA590708
ZA_NCHE_02	2015NCHE002	Zambia	-9.333000	28.716700	VGL	SAMN13337367	PRJNA590708
ZA_NCHE_03	2015NCHE003	Zambia	-9.33300	28.71670	VGL	SAMN13337368	PRJNA590708
ZA_NCHE_04	2015NCHE004	Zambia	-9.33300	28.71670	VGL	SAMN13337369	PRJNA590708
ZA_NCHE_05	2015NCHE005	Zambia	-9.33300	28.71670	VGL	SAMN13337370	PRJNA590708
ZA_NCHE_06	2015NCHE006	Zambia	-9.333000	28.716700	VGL	SAMN13337371	PRJNA590708
MG_AMB_013	2018-AMB-0013	Madagascar	-21.34620	47.71960	VGL	SAMN35113467	PRJNA971625
MG_AND_030	2018-AND-0030	Madagascar	-21.34620	47.69590	VGL	SAMN35113468	PRJNA971625
MG_AND_045	2018-AND-0045	Madagascar	-21.34620	47.69590	VGL	SAMN35113469	PRJNA971625
MG_AND_065	2018-ANDa-0065	Madagascar	-21.34620	47.69590	VGL	SAMN35113470	PRJNA971625
MG_AND_073	2018-ANDa-0073	Madagascar	-21.34620	47.69590	VGL	SAMN35113471	PRJNA971625
MG_ATR_008	2018-ATR-0008	Madagascar	-21.34600	47.77050	VGL	SAMN35113472	PRJNA971625
MG_ATR_009	2018-ATR-009L	Madagascar	-21.34600	47.77050	VGL	SAMN35113473	PRJNA971625
MG_KLL_003	2018-KLL-003L	Madagascar	-21.28120	47.56020	VGL	SAMN19188276	PRJNA729913
MG_KLL_006	2018-KLL-006L	Madagascar	-21.28120	47.56020	VGL	SAMN19188277	PRJNA729913
MG_KLL_007	2018-KLL-007L	Madagascar	-21.28120	47.56020	VGL	SAMN19188278	PRJNA729913
MG_KLL_008	2018-KLL-008L	Madagascar	-21.28120	47.56020	VGL	SAMN19188279	PRJNA729913
MG_KLL_009	2018-KLL-009L	Madagascar	-21.28120	47.56020	VGL	SAMN19188280	PRJNA729913
MG_KLL_010	2018-KLL-010L	Madagascar	-21.28120	47.56020	VGL	SAMN19188281	PRJNA729913
MG_KLL_011	2018-KLL-011L	Madagascar	-21.28120	47.56020	VGL	SAMN19188282	PRJNA729913
MG_KLL_012	2018-KLL-012L	Madagascar	-21.28120	47.56020	VGL	SAMN19188283	PRJNA729913
MG_KLL_013	2018-KLL-013L	Madagascar	-21.28120	47.56020	VGL	SAMN19188284	PRJNA729913
MG_KLL_019	2018-KLL-019L	Madagascar	-21.28120	47.56020	VGL	SAMN19188285	PRJNA729913
ML_BAN_115	93BANA115	Mali	12.800000	-8.050000	VGL	SAMN35108701	PRJNA971624
ML_BAN_237	93BANA237	Mali	12.800000	-8.050000	VGL	SAMN35108702	PRJNA971624
ML_BAN_266	93BANA266	Mali	12.800000	-8.050000	VGL	SAMN35108703	PRJNA971624
UG_AC_093	AC0093-C	Uganda	0.77	34.026	Ag1000G	ERS223969	PRJEB1670
UG_AC_098	AC0098-C	Uganda	0.77	34.026	Ag1000G	ERS223928	PRJEB1670
UG_AC_129	AC0129-C	Uganda	0.77	34.026	Ag1000G	ERS223968	PRJEB1670
UG_AC_138	AC0138-C	Uganda	0.77	34.026	Ag1000G	ERS223865	PRJEB1670
UG_AC_144	AC0144-C	Uganda	0.77	34.026	Ag1000G	ERS223851	PRJEB1670
UG_AC_147	AC0147-C	Uganda	0.77	34.026	Ag1000G	ERS223889	PRJEB1670
UG_AC_148	AC0148-C	Uganda	0.77	34.026	Ag1000G	ERS223904	PRJEB1670
UG_AC_150	AC0150-C	Uganda	0.77	34.026	Ag1000G	ERS223885	PRJEB1670
UG_AC_152	AC0152-C	Uganda	0.77	34.026	Ag1000G	ERS223870	PRJEB1670
UG_AC_160	AC0160-C	Uganda	0.77	34.026	Ag1000G	ERS223880	PRJEB1670

UG_AC_184	AC0184-C	Uganda	0.77	34.026	Ag1000G	ERS223820	PRJEB1670
UG_AC_187	AC0187-C	Uganda	0.77	34.026	Ag1000G	ERS223764	PRJEB1670
UG_AC_197	AC0197-C	Uganda	0.77	34.026	Ag1000G	ERS223839	PRJEB1670
UG_AC_199	AC0199-C	Uganda	0.77	34.026	Ag1000G	ERS223753	PRJEB1670
UG_AC_202	AC0202-C	Uganda	0.77	34.026	Ag1000G	ERS223833	PRJEB1670
CM_AN_0023	AN0023-C	Cameroon	5.747	14.442	Ag1000G	ERS224558	PRJEB1670
CM_AN_0024	AN0024-C	Cameroon	5.747	14.442	Ag1000G	ERS224453	PRJEB1670
CM_AN_0026	AN0026-C	Cameroon	5.747	14.442	Ag1000G	ERS224626	PRJEB1670
CM_AN_0034	AN0034-C	Cameroon	5.747	14.442	Ag1000G	ERS224574	PRJEB1670
CM_AN_0061	AN0061-C	Cameroon	5.747	14.442	Ag1000G	ERS224594	PRJEB1670
CM_AN_0062	AN0062-C	Cameroon	5.747	14.442	Ag1000G	ERS224465	PRJEB1670
CM_AN_0063	AN0063-C	Cameroon	5.747	14.442	Ag1000G	ERS224515	PRJEB1670
CM_AN_0064	AN0064-C	Cameroon	5.747	14.442	Ag1000G	ERS224618	PRJEB1670
CM_AN_0105	AN0105-C	Cameroon	5.747	14.442	Ag1000G	ERS224374	PRJEB1670
CM_AN_0108	AN0108-C	Cameroon	5.747	14.442	Ag1000G	ERS224473	PRJEB1670
CM_AN_0121	AN0121-C	Cameroon	5.747	14.442	Ag1000G	ERS224542	PRJEB1670
CM_AN_0123	AN0123-C	Cameroon	4.341	13.558	Ag1000G	ERS224835	PRJEB1670
CM_AN_0124	AN0124-C	Cameroon	4.341	13.558	Ag1000G	ERS224438	PRJEB1670
CM_AN_0125	AN0125-C	Cameroon	4.341	13.558	Ag1000G	ERS224592	PRJEB1670
CM_AN_0126	AN0126-C	Cameroon	4.341	13.558	Ag1000G	ERS224595	PRJEB1670
CM_AN_0222	AN0222-C	Cameroon	4.777	13.844	Ag1000G	ERS224525	PRJEB1670
CM_AN_0225	AN0225-C	Cameroon	4.777	13.844	Ag1000G	ERS224516	PRJEB1670
CM_AN_0226	AN0226-C	Cameroon	4.777	13.844	Ag1000G	ERS224638	PRJEB1670
CM_AN_0228	AN0228-C	Cameroon	4.777	13.844	Ag1000G	ERS224839	PRJEB1670
MY_AP_002	AP0002-C	Mayotte	-12.73781	45.14170	Ag1000G	ERZ1696325	PRJEB1670
MY_AP_005	AP0005-C	Mayotte	-12.73781	45.14170	Ag1000G	ERZ1696326	PRJEB1670
MY_AP_006	AP0006-C	Mayotte	-12.73781	45.14170	Ag1000G	ERZ1696327	PRJEB1670
MY_AP_007	AP0007-C	Mayotte	-12.70271	45.08109	Ag1000G	ERZ1696328	PRJEB1670
MY_AP_008	AP0008-C	Mayotte	-12.77870	45.14291	Ag1000G	ERZ1696329	PRJEB1670
MY_AP_009	AP0009-C	Mayotte	-12.99066	45.15567	Ag1000G	ERZ1696330	PRJEB1670
MY_AP_010	AP0010-C	Mayotte	-12.79653	45.12172	Ag1000G	ERZ1696331	PRJEB1670
MY_AP_011	AP0011-C	Mayotte	-12.79653	45.12172	Ag1000G	ERZ1696332	PRJEB1670
MY_AP_013	AP0013-C	Mayotte	-12.99066	45.15567	Ag1000G	ERZ1696333	PRJEB1670
MY_AP_014	AP0014-C	Mayotte	-12.99066	45.15567	Ag1000G	ERZ1696334	PRJEB1670
MY_AP_017	AP0017-C	Mayotte	-12.85215	45.10389	Ag1000G	ERZ1696335	PRJEB1670
MY_AP_019	AP0019-C	Mayotte	-12.85215	45.10389	Ag1000G	ERZ1696336	PRJEB1670
MY_AP_020	AP0020-C	Mayotte	-12.85215	45.10389	Ag1000G	ERZ1696337	PRJEB1670
MY_AP_021	AP0021-C	Mayotte	-12.85215	45.10389	Ag1000G	ERZ1696338	PRJEB1670
MY_AP_022	AP0022-C	Mayotte	-12.85215	45.10389	Ag1000G	ERZ1696339	PRJEB1670
MY_AP_023	AP0023-C	Mayotte	-12.85215	45.10389	Ag1000G	ERZ1696340	PRJEB1670
MY_AP_025	AP0025-C	Mayotte	-12.85215	45.10389	Ag1000G	ERZ1696341	PRJEB1670
MY_AP_030	AP0030-C	Mayotte	-12.85700	45.15518	Ag1000G	ERZ1696342	PRJEB1670

MY_AP_031	AP0031-C	Mayotte	-12.85700	45.15518	Ag1000G	ERZ1696343	PRJEB1670
MY_AP_032	AP0032-C	Mayotte	-12.85700	45.15518	Ag1000G	ERZ1696344	PRJEB1670
MY_AP_033	AP0033-C	Mayotte	-12.79653	45.12172	Ag1000G	ERZ1696345	PRJEB1670
MY_AP_034	AP0034-C	Mayotte	-12.79653	45.12172	Ag1000G	ERZ1696346	PRJEB1670
MY_AP_035	AP0035-C	Mayotte	-12.79653	45.12172	Ag1000G	ERZ1696347	PRJEB1670
TZ_BL_046	BL0046-C	Tanzania	-1.96200	31.62100	Ag1000G	ERZ1697469	PRJEB1670
TZ_BL_052	BL0052-C	Tanzania	-1.96200	31.62100	Ag1000G	ERZ1697474	PRJEB1670
TZ_BL_054	BL0054-C	Tanzania	-1.96200	31.62100	Ag1000G	ERZ1697475	PRJEB1670
TZ_BL_058	BL0058-C	Tanzania	-1.96200	31.62100	Ag1000G	ERZ1697477	PRJEB1670
TZ_BL_0346	BL0346-C	Tanzania	-1.96200	31.62100	Ag1000G	ERZ1697726	PRJEB1670
TZ_BL_0347	BL0347-C	Tanzania	-1.96200	31.62100	Ag1000G	ERZ1697727	PRJEB1670
TZ_BL_0348	BL0348-C	Tanzania	-1.96200	31.62100	Ag1000G	ERZ1697728	PRJEB1670
TZ_BL_350	BL0350-C	Tanzania	-1.96200	31.62100	Ag1000G	ERZ1697729	PRJEB1670
TZ_BL_351	BL0351-C	Tanzania	-1.96200	31.62100	Ag1000G	ERZ1697730	PRJEB1670
TZ_BL_352	BL0352-C	Tanzania	-1.96200	31.62100	Ag1000G	ERZ1697731	PRJEB1670
TZ_BL_353	BL0353-C	Tanzania	-1.96200	31.62100	Ag1000G	ERZ1697732	PRJEB1670
TZ_BL_354	BL0354-C	Tanzania	-1.96200	31.62100	Ag1000G	ERZ1697733	PRJEB1670
TZ_BL_356	BL0356-C	Tanzania	-1.96200	31.62100	Ag1000G	ERZ1697734	PRJEB1670
MZ_BQ_046	BQ0046-C	Mozambique	-23.71600	35.29900	Ag1000G	ERZ1697395	PRJEB1670
MZ_BQ_047	BQ0047-C	Mozambique	-23.71600	35.29900	Ag1000G	ERZ1697396	PRJEB1670
MZ_BQ_049	BQ0049-C	Mozambique	-23.71600	35.29900	Ag1000G	ERZ1697397	PRJEB1670
MZ_BQ_050	BQ0050-C	Mozambique	-23.71600	35.29900	Ag1000G	ERZ1697398	PRJEB1670
MZ_BQ_051	BQ0051-C	Mozambique	-23.71600	35.29900	Ag1000G	ERZ1697399	PRJEB1670
MZ_BQ_0052	BQ0052-C	Mozambique	-23.71600	35.29900	Ag1000G	ERZ1697400	PRJEB1670
MZ_BQ_0056	BQ0056-C	Mozambique	-23.71600	35.29900	Ag1000G	ERZ1697404	PRJEB1670
MZ_BQ_080	BQ0080-C	Mozambique	-23.71600	35.29900	Ag1000G	ERZ1697426	PRJEB1670
MZ_BQ_081	BQ0081-C	Mozambique	-23.71600	35.29900	Ag1000G	ERZ1697427	PRJEB1670
MZ_BQ_0082	BQ0082-C	Mozambique	-23.71600	35.29900	Ag1000G	ERZ1697428	PRJEB1670
MZ_BQ_0083	BQ0083-C	Mozambique	-23.71600	35.29900	Ag1000G	ERZ1697429	PRJEB1670
MZ_BQ_0084	BQ0084-C	Mozambique	-23.71600	35.29900	Ag1000G	ERZ1697430	PRJEB1670
MZ_BQ_0085	BQ0085-C	Mozambique	-23.71600	35.29900	Ag1000G	ERZ1697431	PRJEB1670
MZ_BQ_0121	BQ0121-C	Mozambique	-23.71600	35.29900	Ag1000G	ERZ1697462	PRJEB1670
MZ_BQ_0122	BQ0122-C	Mozambique	-23.71600	35.29900	Ag1000G	ERZ1697463	PRJEB1670
MZ_BQ_0123	BQ0123-C	Mozambique	-23.71600	35.29900	Ag1000G	ERZ1697464	PRJEB1670
MZ_BQ_125	BQ0125-C	Mozambique	-23.71600	35.29900	Ag1000G	ERZ1697465	PRJEB1670
MZ_BQ_126	BQ0126-C	Mozambique	-23.71600	35.29900	Ag1000G	ERZ1697466	PRJEB1670
MZ_BQ_129	BQ0129-C	Mozambique	-23.71600	35.29900	Ag1000G	ERZ1697467	PRJEB1670
MZ_BQ_130	BQ0130-C	Mozambique	-23.71600	35.29900	Ag1000G	ERZ1697468	PRJEB1670

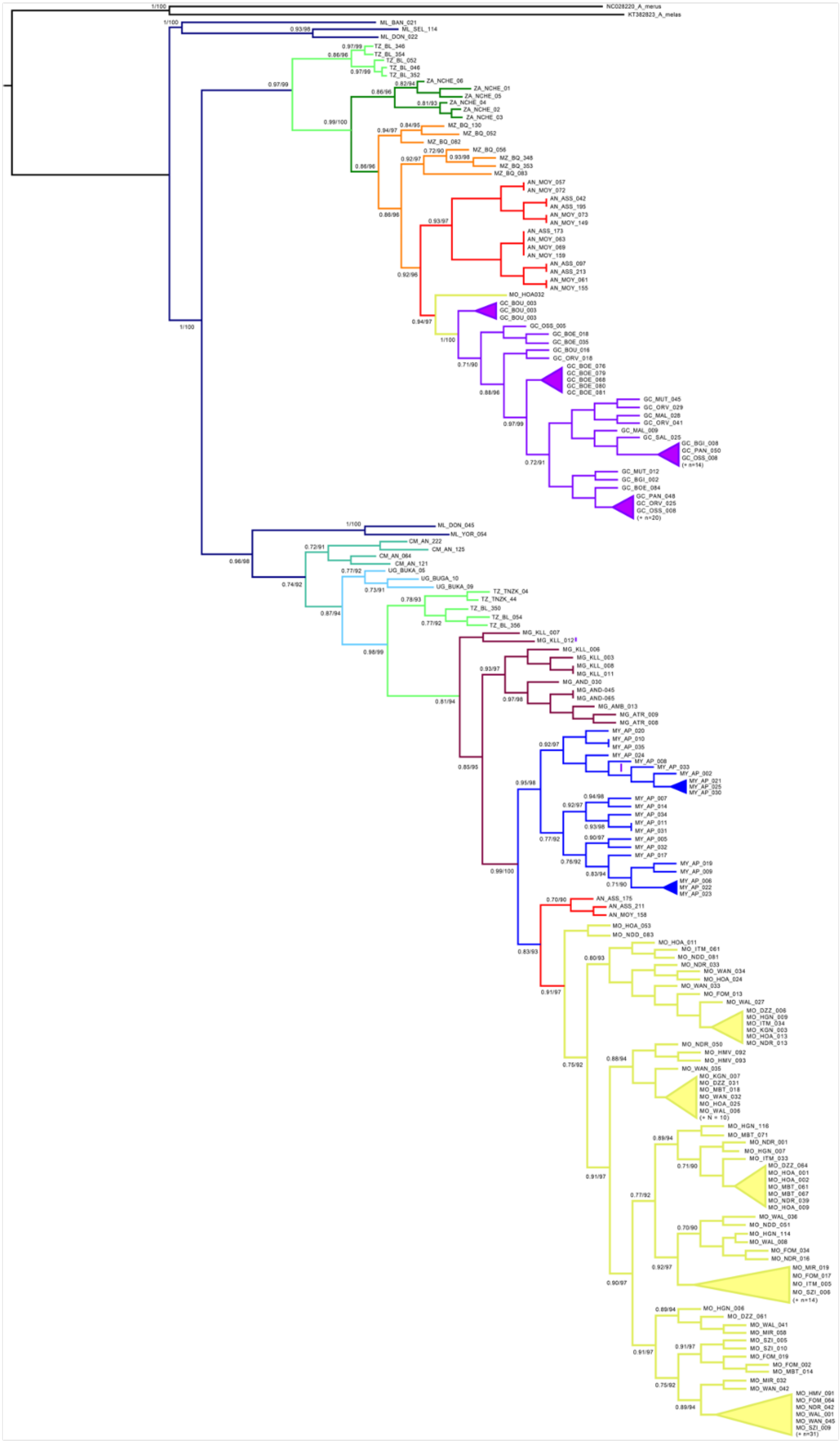

Figure S1 Maximum Likelihood phylogram of the complete set of *Anopheles gambiae*. All nodes recovered with >70 support values are displayed.
